# Looming-eyes buoys fail to reduce seabird bycatch in the Icelandic lumpfish fishery: depth-based fishing restrictions are an alternative

**DOI:** 10.1098/rsos.230783

**Published:** 2023-10-25

**Authors:** Yann Rouxel, Hólmfríður Arnardóttir, Steffen Oppel

**Affiliations:** ^1^ BirdLife International Marine Programme, the Royal Society for the Protection of Birds Scotland, Glasgow, UK; ^2^ Fuglavernd BirdLife Iceland, Reykjavik, Iceland; ^3^ RSPB Centre for Conservation Science, Royal Society for the Protection of Birds, The Lodge, Sandy, UK

**Keywords:** bycatch mitigation, gillnet, fishing closure, Marine Stewardship Council, guillemot

## Abstract

Bycatch in gillnets from lumpfish (*Cyclopterus lumpus*) fisheries is an important conservation issue in the north Atlantic, with up to 30 000 seabirds potentially killed each year. To date, no technical solutions exist to reduce the bycatch of seabirds in gillnet fisheries, but research on above-water bird deterrents as a form of bycatch mitigation has shown promising results. Here, we tested whether a floating device called ‘looming-eyes buoy’ (LEB) would consistently reduce the bycatch of seabirds in the Icelandic lumpfish fishery. We conducted 61 controlled trials with sets of normal gillnets and experimental nets equipped with LEBs. We compared both fish catch and bycatch between net types while accounting for exposure time, water depth and season, and found no effect of LEBs on both target lumpfish catch and bycatch. Our analysis indicated however a strong correlation between bycatch rates and fishing depths, suggesting that depth-based fishing restrictions could virtually eliminate the bycatch of seabirds in this fishery. We estimated that limiting fishing to waters more than 50 m deep could save between 5000 and 9300 seabirds every year, arrest the population decline of endangered black guillemots in Iceland, while having only a marginal effect on target fish catch.

## 1. Introduction

Bycatch from commercial fisheries has been recognized as one of the top three threats affecting seabird species globally [1,2], possibly killing up to 1 million seabirds each year [3]. Nearly half of this estimated figure is attributed to gillnet fisheries alone, with the north Atlantic area thought to be the largest contributor globally [4]. Gillnets commonly refer to a single wall of netting anchored on the seabed, catching fish when they come in contact with the net [5]. Besides seabirds, gillnets are known to be responsible for the death of a large array of non-targeted marine organisms—including marine mammals, turtles and elasmobranchs [6–8].

In the north Atlantic, gillnet fisheries targeting lumpfish *Cyclopterus lumpus* (also referred to as lumpsucker) exist across most of the species' geographical range, spanning from Newfoundland (Canada) in the west, to the Barents Sea in the east and Denmark in the south [9]. The lumpfish is a demersal solitary species living in temperate and cold waters, migrating in spring from their offshore feeding areas to the coastal areas where they remain for several months before spawning [10]. Among the nations involved in north Atlantic lumpfish fishing, Iceland and Greenland account for the vast majority of the landings, with fishing fleets consisting of small coastal vessels (generally less than 15 m in length) using large-mesh bottom set gillnets [11]. Although a small market for male lumpfish exists, which typically represents less than 1% of landings in Iceland, the lumpfish fishery is largely targeting gravid females for their eggs [12,13]. When we refer to the lumpfish fishery in this paper, we will only refer to the fishery targeting gravid females.

This fishery has been identified as having globally important bycatch levels, posing a significant risk to several populations of diving seabirds [9,14–16]. In Iceland alone, which holds the most comprehensive data for this type of fishery, over 8000 seabirds are estimated to be killed each year [9]. This includes around 4000 common eiders (*Somateria mollissima*), approximately 1800 black guillemots (*Cepphus grylle*), approximately 1200 common guillemots (*Uria aalge*) and nearly 900 great cormorants/European shags (*Phalacrocorax carbo* and *Phalacrocorax aristotelis*), as well as smaller levels of bycatch of other seabird species. The bycaught seabird species are of serious conservation concern, with—among the most commonly caught species—Black guillemot listed as 'Endangered' and three other species listed as 'Vulnerable' under the Icelandic Red list for birds 2018. Among the less frequently caught species [9] two are listed Near Threatened, three as Vulnerable, one as Endangered (Brünnich's guillemot *Uria lomvia*) and one as Critically Endangered (Atlantic puffin *Fratercula arctica*). In the west Greenland lumpfish fishery, between 10 000 and 20 000 seabirds could be bycaught annually [16]. Although bycatch estimates from Norwegian and Danish lumpfish fisheries are lower, the self-reporting nature of bycatch data and the low coverage of fishing effort are most likely underestimating the true scale of bycatch in those countries [9]. In the Icelandic lumpfish fishery, bycatch rates reported by inspectors were five times higher when compared with self-reporting logbooks [17] and equally five times higher when compared with Norwegian and Danish fisheries [9].

Fishing restrictions have been adopted in Iceland to limit the bycatch of marine megafauna. Since 2020, several areas in northwestern Iceland were closed to fishing to reduce the risk of marine mammal bycatch. In addition, setting nets within the vicinity of eider duck colonies without the permission of the colony's owner is prohibited during certain times of the year (Regulation 165/2020). Owing to the high bycatch levels of endangered, threatened and protected species, the Icelandic lumpfish gillnet fishery was suspended from its Marine Stewardship Council (MSC) certification in 2018, and ultimately withdrawn from the programme [18]. The fishery was re-certified in 2020 under conditions to reduce the bycatch of endangered, threatened and protected species, in particular harbour seals (*Phoca vitulina*) and black guillemots [19]. There is therefore an urgent need to develop solutions to reduce bycatch in this fishery.

Various technical mitigation measures have been developed and have proved effective in reducing seabird bycatch in longline and trawl fisheries [20,21], but no ubiquitously effective solution has so far been found to reduce seabird bycatch in gillnet fisheries. Previous attempts to make gillnets more visible underwater through high-contrast panels or LED lights had very contrasting results across species and fisheries [22–25], and sometimes reduced fish catch as well as bycatch [26,27]. A modified standard lumpfish gillnet, consisting of the addition of a 45 cm high small-meshed net panel to the bottom part of the net, has been tested in the west Greenland lumpfish fishery [27]. While bycatch of common eider was significantly reduced, target fish catch was also significantly impacted.

Visually deterring seabirds from diving in the vicinity of gillnets using surface visual signals has been suggested as a new approach to bycatch mitigation in gillnet fisheries, culminating in the testing of stationary kites attached to buoys that resemble birds of prey [28] and ‘looming-eyes buoys' (LEBs), a buoy with a rotating set of looming black-and-white circles which superficially resemble the staring eyes of a predator [29]. In an experimental non-commercial setting in Estonia, the LEB device significantly reduced the presence of long-tailed ducks from a 50 m radius [29], and could in theory significantly reduce seabird bycatch when deployed at the surface of a net.

Here, we test whether the LEB device actually reduces seabird bycatch rates in the Icelandic lumpfish fishery, and whether it has any effect on target lumpfish catch. We conducted a controlled trial study and accounted for temporal and spatial variation in seabird bycatch and fish catch rates when evaluating the effectiveness of the LEB. Based on our results, we derive recommendations to how the Icelandic lumpfish fishery could reduce bycatch of both seabirds and marine mammals.

## 2. Methods

### 2.1. Study area and fishing characteristics

**Figure 1.**
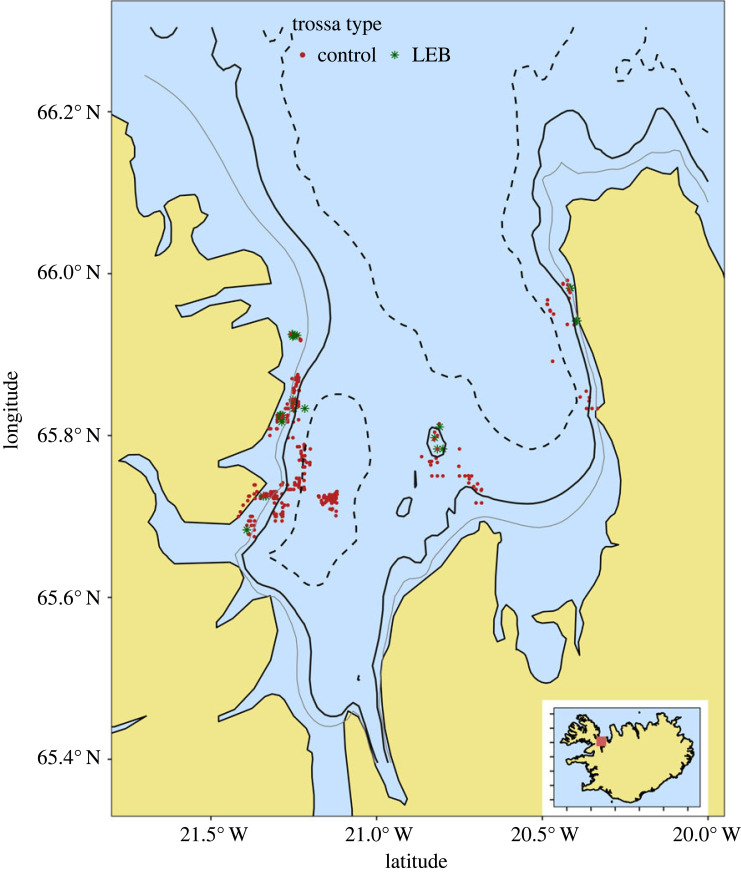
Location of the at-sea trials of looming-eyes buoys (LEB; green dots) and control fishing nets (red dots) conducted during this project in the Icelandic lumpfish fishery in northwest Iceland. Contour lines indicate 25 m (light grey) and 50 m (dark grey) and 100 m depth (dashed).

Our experiment took place in the Húnaflói Bay in northern Iceland, located between the Westfjords and Skagafjörður (65°48'30.0″ N, 20°53'03.7″ W; figure 1). This large bay is roughly 50 km wide and 100 km long, and is one of the most important areas for lumpfish fishing in Iceland [30,31]. We conducted our experiment between 21 March and 18 May 2022, and a total of seven fishing boats participated. Under the current regulation (no. 165/2020), each boat is allowed to deploy a total of 7500 m of nets and to leave them unattended for a maximum of 3 days, unless adverse weather conditions prevent safe net recovery [13]. Each boat deploys a number of ‘tiers' of net (individual nets are tied together to make a tier) locally known as ‘trossa', which are typically 615 m long, and generally set between 5 and 50 m deep. Trossa is the unit of effort we used in this project, with participating boats using 11 trossas on average (range 5–16).

### 2.2. Looming eyes buoy

**Figure 2.**
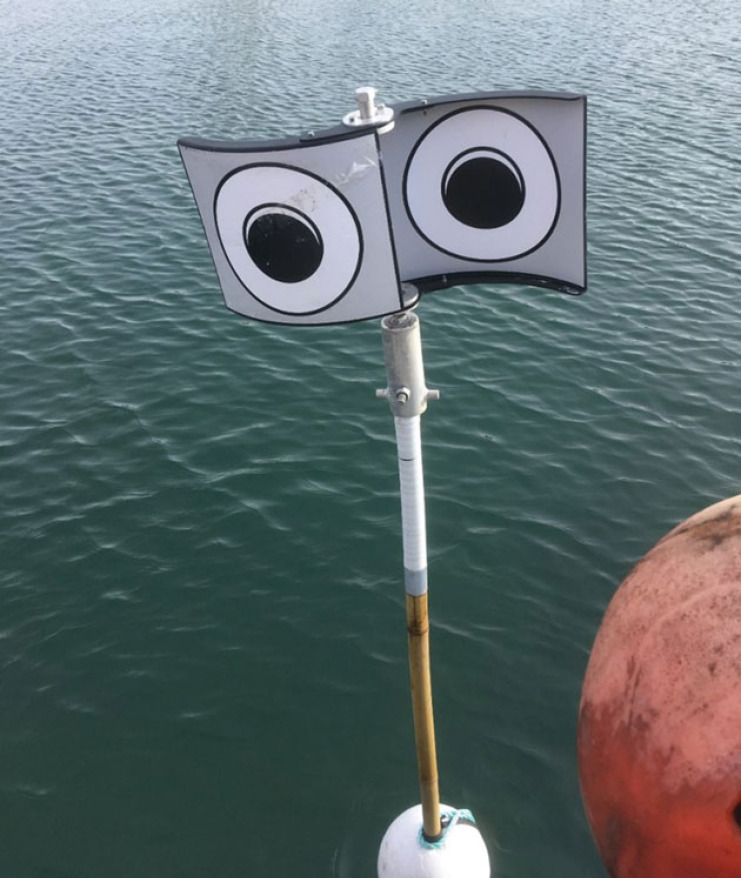
Model of looming-eyes buoy tested in this study. Photo credit: Paul St Pierre, RSPB.

We upgraded the LEB tested in Rouxel *et al*. [29], in collaboration with Fishtek Marine Ltd, to be deployed by fishers during commercial fishing conditions. The device was assembled around a 3200 mm long bamboo pole, with a 5 kg concrete counter-weight at the bottom end, a torpedo shaped polystyrene buoy at the centre, and with rotating panels at the top (figure 2). Each individual rotating panel measured 260 mm in length and 221 mm in height, made of 4 mm thick black acrylonitrile butadiene styrene. They were shaped similar to a sinusoidal wind turbine to facilitate movement by the wind. Natural wind gusts induced unpredictable movements and speed rotations, which further intensify the likelihood of birds' behavioural responses and reduce chances of habituation [29]. The two faces of each panel exhibited an eye pattern of different size, to create the ‘looming' effect when the panels rotate. The panels were designed to ensure they could be detected from a distance of at least 50 m even during relatively low light levels.

### 2.3. Experimental design

In this experiment, fishers' were asked to use one of their trossas as ‘experimental', with LEBs deployed across its length. One LEB was deployed at each extremity of the trossa, and every 100 m, meaning that 5–6 LEBs were deployed along each experimental trossa on average. The other normal-trossas were considered as ‘control'. Seabird and marine mammal bycatch were reported, but also fish catch, fishing characteristics (mesh size, net width and length, number of nets, time of setting and hauling) and environmental variables (latitude, longitude, wind speed, water depth). We used a mix of self-reporting data and on-board observation. All collected data were entered into a database after each fishing trip. Participating fishers followed their normal fishing procedures, to ensure that trials were representative of real fishing conditions in this fishery. To avoid ‘contamination' of control trossas with the presence of nearby LEBs, a minimum distance of 200 m between experimental and control trossas was respected.

### 2.4. Analysis

#### 2.4.1. Quantification of looming eyes buoy effect

Our main goal was to evaluate the LEB as a bycatch reduction measure for seabirds. We therefore first quantified the catch of fish and the bycatch of birds and mammals for each trossa, and expressed the catch or bycatch as the total number of lumpfish, birds (of all species), or marine mammals caught per trossa area (calculated as net length × number of nets × net height) per day as the catch per unit effort (CPUE) and bycatch per unit effort (BPUE), respectively. We did not expect the LEB (as an above-water deterrent device) to affect fish catch or mammal bycatch, but included mammals in our calculations to inform future management recommendations.

We then calculated median and 95% bootstrapped confidence intervals (CIs) by randomly drawing *n* = 61 samples (number of paired trials) with replacement from all trossas with LEBs, and repeating this procedure for 10 000 random draws. Because each LEB trossa was paired in a single fishing trip with 4–16 control trossas, we randomly drew one matching control trossa from the same fishing trip for the comparison during each of the 10 000 random draws. Because water depth was an important factor influencing bycatch (see Results, e.g. electronic supplementary material, figure S1), we first drew a target depth for the control trossa from a random normal distribution with the mean depth at which the LEB trossa was set and a standard deviation of 5 m, and then selected the control trossa that was set at a depth closest to the target depth. We quantified the effect size of the LEB as the difference in the number of bycaught birds per trossa day from the median of 10 000 bootstrap samples, and calculated the 95% CIs as the 2.5% and 97.5% quantiles of the 10 000 samples. If the CI of the difference overlapped zero, we concluded that the effect of the LEB was not statistically significant to reduce bird bycatch in Icelandic lumpfish gillnets. We used a similar procedure to estimate the effect of LEBs on target fish catch, for marine mammals, and for selected species for which sufficient data were available (e.g. common eider, guillemots and long-tailed ducks *Clangula hyemalis*). To present results, we extrapolated the median catch or bycatch rate to the average size of a trossa (2000 m^2^, range: 993.2–2837.7 m^2^) and present estimates as median per trossa day.

### 2.5. Predictors in variation of bycatch

Bycatch of seabirds or mammals may be affected by many environmental variables, such as time of year, distance from the coast, or water depth [32]. To explore whether the LEB could explain any variation in either the occurrence or the abundance of bycatch given that other environmental variables may have affected the recorded bycatch, we used a powerful multivariate random forest algorithm [33–35]. We aimed to explain the occurrence of bycatch and the number of seabirds bycaught by eight variables that could plausibly affect seabird bycatch: fishing trip (to account for individual variation among boats and fishermen), distance to the coast, water depth at which nets are set, area of the trossa, soaking time (in days), day of the year (to account for seasonal variation), and the presence of LEBs. A random forest is a machine-learning algorithm based on ensembles of regression trees that can accommodate many predictor variables and yields highly accurate predictions, while accounting for complex interactions. We therefore expected that the random forest model would identify if the LEB might explain variation in bycatch only under certain environmental conditions. We implemented the model in the R package ‘randomForest' [36], and evaluated the explanatory power of the random forest model by calculating what proportion of bycatch events were accurately classified (in the occurrence model), and what proportion of the variation in bycatch numbers was explained (in the abundance model).

To quantify the relative importance of predictor variables on our response variables, and assess whether the presence of an LEB was an important predictor of bycatch occurrence or abundance, we used a permutation procedure that calculates the loss in predictive accuracy of the random forest model after randomly permuting a given variable [37–39]. We implemented this assessment using the R function ‘varImp' and present results as relative variable importance, with the most important variable (greatest reduction in accuracy after permutation) assigned a value of 100%.

### 2.6. Exploring alternative ways of bycatch reduction

After having determined the relative importance of variables predicting seabird bycatch, we explored whether management scenarios could be designed to reduce overall bycatch levels in the Icelandic lumpfish fishery. Specifically, we explored whether depth-based fishing restrictions could maintain target catch quotas but reduce bycatch.

To estimate the potential effect of a depth-based fishing restriction, we first estimated the average seabird bycatch and target fish catch rates per 10 m water depth interval from all of our data. We then used the Icelandic lumpfish landing statistics [13,40] to estimate what proportion of the total lumpfish landings were derived from fishing at certain depths (in 10 m intervals). Lumpfish landing statistics were available from 2014 to 2021 and ranged between 4516 tons (2018) and 7601 tons (2021) per year. We assumed that the average female lumpfish weighs 3 kg to convert reported landings (in tons) into number of lumpfish [30,41,42]. We used these calculated numbers of caught lumpfish and our empirically derived target fish catch rates at each depth interval to calculate the mean and 95% CIs of fishing effort in each 10 m depth interval from 2014 to 2021. This fishing effort was then multiplied with the depth-specific seabird bycatch rates to yield the annual total bycatch across the Icelandic lumpfish fishery, a slightly refined extrapolation to the previous approach that did not consider the depth distribution of fishing effort and bycatch rates [9], or the actual effort (trossa size and soak time) of each fishing trip [40].

We then explored whether a restriction that limits fishing in shallow waters could reduce seabird bycatch, and calculated the potential effect on target fish catch. We caution that such an analysis makes several simplifying assumptions, namely that (i) the target catch rates at greater depth can be maintained when a greater proportion of fishing effort occurs at greater depths, and (ii) the economic cost of fishing at greater depth is negligible such that overall fishing effort is maintained at a fleet level. Under these assumptions, we simulated that in every year between 2014 and 2021 fishing would have been closed for water depths shallower than 10, 20, 30, 40 and 50 m. We re-allocated the fishing effort that occurred in the banned depths in equal proportion to the available greater depths to mimic the displacement effect of spatial fishing closures. We then re-calculated the total fish catch and the seabird bycatch under the simulated re-distribution of fishing effort using our empirical depth-specific target catch and seabird bycatch rates as described above. We present the relative potential reduction in seabird bycatch compared to the extrapolated total bycatch, and the relative potential change in target fish catch. We do not present total extrapolated numbers of bycaught marine mammals, because the patterns of mammal bycatch in our study area were not representative to be extrapolated across the Icelandic fishery [40]. Nonetheless, we present the relative potential reduction in marine mammal bycatch to inform potential consequences to regulations aimed at reducing seabird bycatch.

## 3. Results

A total of 84 independent fishing trips were completed during this study, representing data from 936 trossas including 61 experimental trossas with LEBs. Independent observers were deployed onboard 13 of the total fishing trips, representing around 16% of the fishing effort. In total, 250 seabirds were reported bycaught during this experiment (table 1). This included 151 common eiders, 48 common guillemots, 29 black guillemots and 10 long-tailed ducks. Great cormorants (7), European shags (2), Atlantic puffin, northern fulmar and red-throated diver *Gavia stellata* (one each) were also reported as bycatch. No birds were caught in 782 of the 936 trossas (83%), with 84% of the control trossas and 72% of the LEB trossas not registering any bycatch. Besides seabirds, a total of 29 marine mammals were also reported as bycatch (table 1). Bycaught marine mammals included 10 harbour porpoises *Phocoena phocoena*, 10 harbour seals, three grey seals *Halichoerus grypus* and three harp seals *Pagophilus groenlandicus*, as well as two bottlenose dolphins *Tursiops truncatus* and one white-beaked dolphin *Lagenorhynchus albirostris*. A total of 214 seabirds and 24 marine mammals were bycaught in control trossas (*n* = 875), while experimental trossas (*n* = 61) reported 36 seabirds and five marine mammals bycaught. A total of 73 350 lumpfish were reported caught by the participating vessels during this experiment.

**Table 1.** Summary table of reported bycatch during experimental tests of looming-eyes buoys in the Icelandic lumpfish fishery in 2022. (A trossa is a ‘tier' of nets tied together that are stationary at the bottom of the sea for an average duration of 3 days.)

species	control (*n* = 875 trossas)	experimental (*n* = 61 trossas)	total
seabirds			
black guillemot	26	3	29
common guillemot	43	5	48
atlantic puffin	1		1
european shag	2		2
great cormorant	7		7
common eider	124	27	151
long-tailed duck	10		10
red-throated diver	1		1
northern fulmar		1	1
total seabirds	214	36	250
marine mammals			
harbour seal	7	3	10
grey seal	2	1	3
harp seal	3		3
white-beaked dolphin	1		1
bottlenose dolphin	2		2
harbour porpoise	9	1	10
total marine mammals	24	5	29

### 3.1. Quantification of looming eyes buoy effect

**Figure 3.**
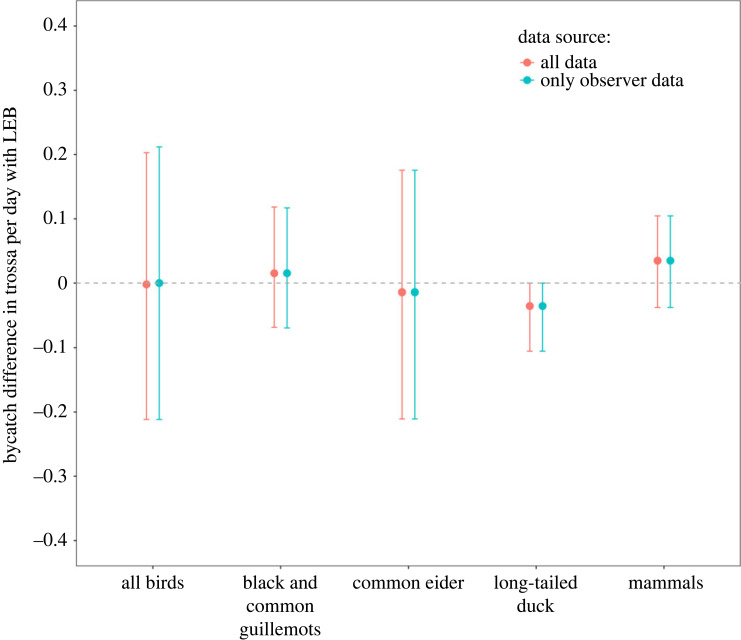
Estimated effect size (median and 95% confidence intervals (CIs)) of the looming eyes buoy (LEB) on the bycatch of several seabird and marine mammals in the Icelandic lumpfish fishery in 2022. Effect size is calculated as the difference in bycatch between a trossa with LEB and a trossa at similar depth on the same fishing trip without LEB. Values less than 0 (dashed line) indicate that bycatch was lower with LEB than without LEB, if CIs overlap with 0 the effect was not significant.

The median bird bycatch was 0.19 birds per trossa day in experimental trossas with LEB, and 0.18 birds per trossa day in control trossas set at a comparable depth (table 2). The median difference therefore included zero and was not statistically significant (table 2 and figure 3). This difference was not affected if only data with an observer on board were used (median −0.008; 95% CI: −0.218–0.201 birds per trossa day). There was also no significant reduction in bycatch for any of the three most commonly caught species (common eiders, common and black guillemots, and long-tailed ducks; table 2 and figure 3), but we note that the sample size for long-tailed ducks was too small to infer the absence of an effect, as no long-tailed ducks were caught in any trossa with an LEB.

**Table 2.** Summary of median catch rate of seabirds, marine mammals and lumpfish in the Icelandic lumpfish fishery in 2022 in trossas with a looming eyes buoy (LEB) and control trossas set at comparable depth during the same fishing trip without LEB. (Median and 95% confidence interval (CI) calculated from 10 000 random bootstrap samples (shown in brackets). Difference is shown in figure 3.)

	median catch per trossa day		
	(with 95% CIs)		
	**experimental (LEB)**	**control**	**difference**
*all birds*	0.19 (0.04–0.36)	0.18 (0.02–0.42)	0 (−0.21–0.21)
common and black guillemot	0.06 (0–0.14)	0.04 (0–0.11)	0.02 (−0.07–0.12)
common eider	0.11 (0–0.28)	0.11 (0–0.3)	−0.01 (−0.21–0.18)
long-tailed duck	0 (0–0)	0.04 (0–0.11)	−0.04 (−0.11–0)
*all marine mammals*	0.03 (0–0.1)	0 (0–0.04)	0.03 (−0.04–0.1)
*lumpfish*	32.42 (21.46–44.02)	27.19 (19.65–35.51)	5.02 (−4.41–15.3)

The median marine mammal bycatch was 0.03 mammals per trossa day in experimental trossas with LEB, and 0 in unmodified control trossas set at a comparable depth (table 2). The median difference was therefore 0.03 mammals per trossa day that were additionally caught in trossas with LEB, but owing to the large variation this effect estimate included zero and was therefore not statistically significant (table 2 and figure 3). The difference was identical if only observer data were used.

The median fish catch was 32 lumpfish per trossa day in experimental trossas with LEB, and 27 in unmodified control trossas set at a comparable depth (table 2). The median difference was therefore five fish per trossa day that were additionally caught in trossas with LEB, but owing to the large variation this effect estimate included zero and was therefore not statistically significant (95% CI: −4.4–15.3). This difference was not affected if only data with an observer on board were used (median 5.1; 95% CI: −4.3–15.4 fish per trossa day).

### 3.2. Predictors in variation of bycatch

To explore whether the LEB could explain the occurrence or abundance of bycatch in a multivariate setting that considered other environmental determinants of bycatch, we fitted four separate random forest models, for occurrence and abundance of bird bycatch (all species combined), with all data or only data obtained by independent observers. All random forest models performed poorly and less than half of all bycatch events were accurately predicted (error rate 78% for all data, 57% for observer data) in the occurrence models. The abundance models explained less than 5% of the variation in the data, and in all models the presence of an LEB was the least important variable (electronic supplementary material, figure S1). Despite the poor predictive performance, the depth of the water appeared to explain most of the predictable variation in the occurrence and abundance of seabird bycatch (electronic supplementary material, figure S1).

### 3.3. Exploring alternative ways of bycatch reduction

**Figure 4.**
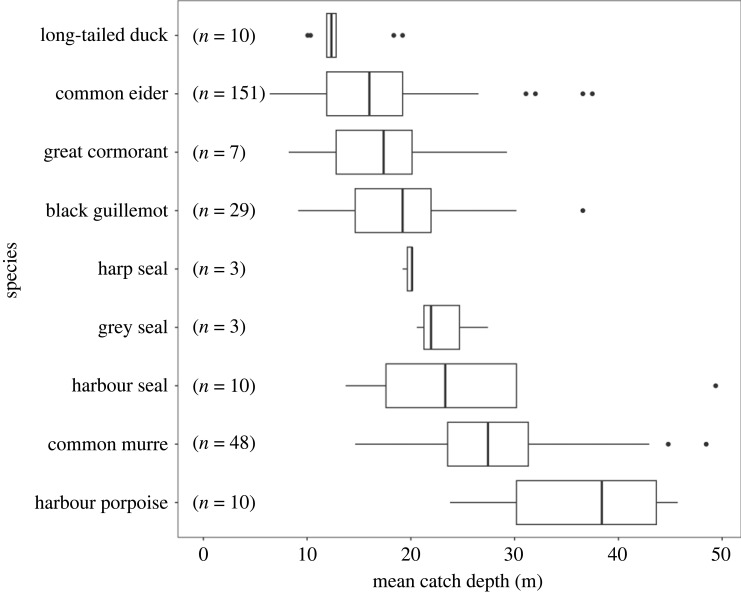
Distribution of the mean sea depth at which different bird and marine mammal species were bycaught in Iceland's lumpfish gillnets in 2022 (shown for those species with a minimum of three individuals caught). Sample size indicates the number of animals that were captured and from which the depth distribution is derived. Boxplot shows the median (vertical bar), inter-quartile range (box), 95% confidence intervals (horizontal bars) and outliers (points).

Because water depth was the most prominent predictor of seabird bycatch with a notable pattern in our data (electronic supplementary material, figure S1), we summarized fish catch and bycatch per average water depth at which trossas were set. Among our experimental fishing trips, greater than 80% of effort was in waters between 10 and 40 m depth (electronic supplementary material, table S1). While marine mammal bycatch was evenly distributed between 10 and 50 m water depth, 90% of the seabird bycatch occurred in waters less than 30 m deep (figure 4; electronic supplementary material, table S1).

Using the depth-specific fish catch rates from our experiment (table 3) and the national fish landing statistics and proportion of national landings from different depths [13,40], we calculated the fishing effort at certain depths for the years 2014–2021. Multiplying the depth-specific fishing effort with our empirically determined depth-specific bycatch rates of seabirds resulted in a mean total annual bycatch of 6572 birds (range 4972–9276; table 3).

**Table 3.** Summary of depth-specific catch rates of fish, seabirds and marine mammals in the Icelandic lumpfish fishery in 2022, and extrapolated total catch and seabird bycatch per depth category averaged over 2014–2021 under the assumption of temporally consistent catch rates. (The proportion of national landings indicates what proportion of the total annual fish catch of the Icelandic lumpfish fishery is caught at the specified depth [13,40].)

depth (m)	fish catch rate (fish per trossa day)	seabird bycatch rate (birds per trossa day)	mammal bycatch rate (mammals per trossa day)	proportion of national landings	average annual fish catch (2014–2021) (tons)	average annual seabird bycatch (2014–2021) (individuals)
0–10	43.03	0.38	0.00	0.13	700.3	2067
10–20	36.13	0.19	0.01	0.34	1,831.5	3204
20–30	32.84	0.09	0.01	0.18	969.6	902
30–40	33.51	0.05	0.02	0.13	700.3	329
40–50	31.41	0.01	0.02	0.1	538.7	70
>50	39.76	0.00	0.00	0.12	646.4	0

**Figure 5.**
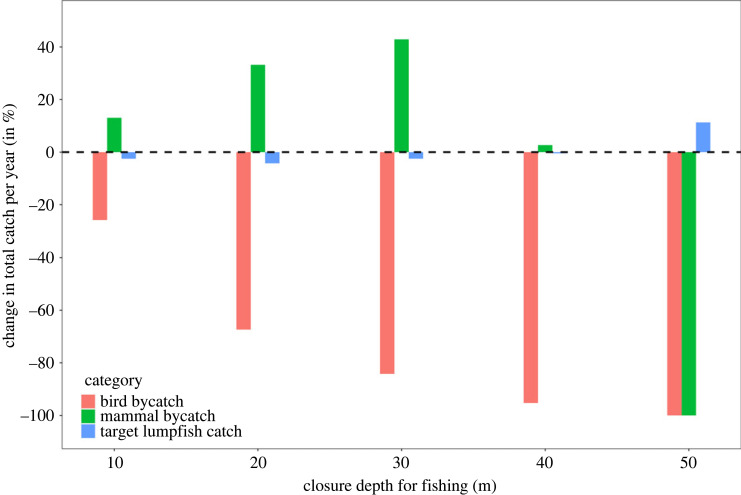
Simulated proportional change of annual total fish catch and seabird and marine mammal bycatch in the Icelandic lumpfish fishery if the fishery was restricted to operate only in waters exceeding a certain depth. See table 4 for absolute annual catch totals for seabirds; for marine mammals the proportional reduction is based on our sampling area and may not be representative across the Icelandic fishery.

By restricting the Icelandic lumpfish fishery to waters exceeding a certain depth, while maintaining and redistributing fishing effort to deeper waters, the overall annual fish catch would remain unaffected around approximately 5200 tons per year (table 4 and figure 5). However, seabird bycatch could be dramatically reduced by greater than 50% if the fishery was limited to waters more than 20 m deep (table 4 and figure 5), potentially saving thousands of seabirds every year. By contrast, bycatch of marine mammals, which were more frequently caught in deeper waters in our study area, would probably increase by up to 43% if the fishery was redistributed to fish in waters more than 30 m deep (table 4 and figure 5). A restriction of the fishery to operate in waters more than 50 m deep would potentially reduce seabird and marine mammal bycatch to near zero while improving target fish catch (table 4 and figure 5)

**Table 4.** Simulated average (and range) annual catch of lumpfish (in tons), and bycatch of seabirds (in individuals) in the Icelandic lumpfish fishery under different scenarios of depth-based fishing restrictions. (Fishing effort above the restriction depth was redistributed to deeper waters, and extrapolated totals are based on reported fish landings between 2014 and 2021 and depth-specific rates of fish catch and bycatch (table 3).)

restriction depth (m)	projected annual total fish catch (tons)	projected annual total seabird bycatch (individuals)
>10	5252 (3972–7410)	4876 (3688–6881)
>20	5157 (3900–7277)	2146 (1623–3028)
>30	5252 (3972–7412)	1038 (785–1465)
>40	5363 (4056–7567)	310 ((235–438)
>50	5996 (4535–8461)	0 (0–0)

## 4. Discussion

We show that the bycatch of seabirds in the Icelandic lumpfish fishery is unlikely to be mitigated by the deployment of LEBs . We found similar seabird bycatch rates for trossas with and without LEBs, including when other environmental variables that are known to influence the probability of seabird bycatch were accounted for. Therefore, we found no evidence that the LEB explained any variation in seabird bycatch. All long-tailed ducks were caught on control trossas during this experiment, which might confirm that LEBs have an effect on the presence of this species, as shown in Rouxel *et al*. [29]. However, owing to the low sample size for this species (10) in Iceland, we cannot infer that the LEB effectively reduces bycatch of long-tailed ducks. Other devices that have deterred birds from fishing operations—such as predator-shaped kites—should continue to be explored in similar experiments to test whether they can actually reduce seabird bycatch in a commercial gillnet fishing operation without affecting fish catch [27,28]. Additionally, a delayed start of the lumpfish fishing season has been identified as a potentially effective mitigation measure to common eider bycatch in Greenland [27].

Because water depth was an important predictor for bycatch, we propose that solutions focussing on water depth restrictions might be more promising to reduce the bycatch of both seabirds and marine mammals in this fishery [43]. Based on our simulations, imposing a type of spatial fishing closure by restricting the Icelandic lumpfish fishery to waters greater than 50 m deep would probably have important benefits for marine megafauna while incurring little to no costs in terms of target catch, a problem found with other mitigation options [26,27].

Our simulations and extrapolations come with important assumptions and limitations that need to be considered when developing regulations. Our study was conducted in a single year and in a single bay in Iceland, and it is unclear whether the bird and mammal bycatch rates that we found in our study are representative across the entire lumpfish fishery in Iceland, because bycatch patterns typically vary in space and time [32]. However, our extrapolations of total annual seabird bycatch are of a similar magnitude as previous extrapolations that were based on an entirely different sampling scheme and did not consider depth-specific seabird bycatch rates [9,40], and we therefore conclude that our seabird bycatch extrapolations are probably reliable. By contrast, a previous estimate on the number of marine mammals being bycaught in this fishery [40] differed from our extrapolations. We caution that the marine mammal bycatch in the bay and year that we studied was lower than the average marine mammal bycatch rate in the lumpfish fishery across all of Iceland. Alternatively, the method of extrapolation may have resulted in different estimates, because the Marine and Freshwater Research Institute (MFRI; [40]) did not have access to fishing effort data (number and size of trossas, soak time). However, even if we replicated the method of extrapolation of MFRI [40], we obtained lower bycatch totals, and we therefore conclude that the different method of extrapolation cannot explain the discrepancies in extrapolated bycatch of marine mammals. Nonetheless, our extrapolated annual catch and bycatch totals for fish and seabirds were of a comparable order of magnitude as previous studies and therefore probably robust.

The different reliability of our extrapolations for seabirds and mammals poses a non-trivial challenge when deciding on regulations. We found that seabird bycatch would generally decrease if fishing was restricted to progressively deeper waters, even if the same amount of fishing effort was displaced from shallower to deeper waters. However, because the mammal bycatch rate in our dataset was actually greatest between 30 and 50 m depth (table 3), a displacement of fishing effort from waters less than 30 m deep to waters between 30 and 50 m deep would actually lead to an increase in marine mammal bycatch (figure 5). By contrast, MFRI [40] found a lower marine mammal bycatch rate in waters more than 30 m deep than in shallower waters, which again highlights that our data may not be representative for marine mammal bycatch in the entire Icelandic lumpfish fishery. We therefore recommend that the depth distribution of marine mammal bycatch is examined in greater detail before depth-specific fishing restrictions could be imposed on the Icelandic lumpfish fishery.

Despite some uncertainty over marine mammal bycatch rates, our seabird bycatch data confirm that the three most bycaught bird species in this fishery are of conservation concern according to the Icelandic and the European Red List. Therefore, ambitious fishery management actions are required to reduce bycatch rates and comply with MSC certification stipulations. A greater than 50 m fishing depth restriction would virtually eliminate bycatch for most seabird species, while a greater than 20 m limit would have a much lesser effect on the bycatch rate of guillemot species, which generally occurred in deeper waters (figure 4). Inadequate measures (e.g. a limited restriction to waters more than 10 m deep) could potentially increase bycatch for common and black guillemots, and potentially marine mammals. Black guillemots are listed as ‘Endangered' under the Icelandic Red list, and are particularly impacted by the lumpfish fishery. The black guillemot population in Iceland has decreased on average by approximately 2% each year between 1981 and 2014, and bycatch from the lumpfish fishery is believed to remove between 6 and 9% of the local population each year (approx. 1800 individuals out of a population of 20 000–30 000 birds; [44,45]). We found that even with a moderate fishing restriction of more than 30 m depth in place, up to 90% of the black guillemot bycatch could be prevented, which may be sufficient to arrest the population decline observed in the last decades. In the light of our findings, a recent study on the foraging behaviour of black guillemots in Norway highlights the importance of shallow marine areas (less than 50 m) for this species, with foraging probability declining with increasing water depth [46].

Although there appears to be a clear benefit of depth-specific fishing restrictions for seabirds and potentially marine mammals, our study cannot evaluate the practical and economic feasibility of fishing in deeper waters. Our data show that the catch rates of lumpfish in waters deeper than 50 m was as great or greater than in shallower waters, but we caution that these extrapolations are based on a small sample as most fishermen prefer to fish in shallower waters. In addition, deeper waters are generally farther from shore, and will therefore require boats to travel farther to reach fishing grounds, although the steep topography around most parts of Iceland is unlikely to lead to prohibitive fuel consumption if fishing was restricted to waters more than 50 m deep. If, however, fishing effort is displaced from shallow to deeper waters, competition between fishers may increase. We therefore recommend consultation with fishers to understand the social and economic consequences of imposing depth-specific fishery regulations to ensure that those regulations are supported by and adhered to by the community of lumpfish fishers.

In summary, we show that LEBs were not effective at reducing seabird bycatch in the Icelandic lumpfish fishery. However, as an immediately available alternative solution, depth-based fishing restrictions appear to have a great potential to significantly reduce seabird bycatch rates with marginal effect on target fish catch. While the practicality and economic viability of such restrictions need to be refined, depth-based fishing restrictions are currently the only known measures that could significantly reduce seabird bycatch levels in this type of fishery.

## Data Availability

The database and statistical analysis can be accessed via the following reference and link: Rouxel Y, Oppel S. 2023 Iceland lumpfish 2022 bycatch mitigation trials (dataset) *Zenodo* (https://doi.org/10.5281/zenodo.8422595) [47]. Please also see the electronic supplementary material for additional table and figures [48].
